# Elevated paternal glucocorticoid exposure alters the small noncoding RNA profile in sperm and modifies anxiety and depressive phenotypes in the offspring

**DOI:** 10.1038/tp.2016.109

**Published:** 2016-06-14

**Authors:** A K Short, K A Fennell, V M Perreau, A Fox, M K O'Bryan, J H Kim, T W Bredy, T Y Pang, A J Hannan

**Affiliations:** 1 Behavioural Neuroscience Division, Florey Institute of Neuroscience and Mental Health, Melbourne Brain Centre, University of Melbourne, Parkville, VIC, Australia; 2 Department of Anatomy and Neuroscience, University of Melbourne, Parkville, VIC, Australia; 3 Department of Anatomy and Developmental Biology, The Development and Stem Cell Program, Monash Biomedicine Discovery Institute, Monash University, Melbourne, VIC, Australia; 4 Cognitive Neuroepigenetics Group, Queensland Brain Institute, The University of Queensland, St Lucia, QLD, Australia

## Abstract

Recent studies have suggested that physiological and behavioral traits may be transgenerationally inherited through the paternal lineage, possibly via non-genomic signals derived from the sperm. To investigate how paternal stress might influence offspring behavioral phenotypes, a model of hypothalamic–pituitary–adrenal (HPA) axis dysregulation was used. Male breeders were administered water supplemented with corticosterone (CORT) for 4 weeks before mating with untreated female mice. Female, but not male, F1 offspring of CORT-treated fathers displayed altered fear extinction at 2 weeks of age. Only male F1 offspring exhibited altered patterns of ultrasonic vocalization at postnatal day 3 and, as adults, showed decreased time in open on the elevated-plus maze and time in light on the light–dark apparatus, suggesting a hyperanxiety-like behavioral phenotype due to paternal CORT treatment. Interestingly, expression of the paternally imprinted gene *Igf2* was increased in the hippocampus of F1 male offspring but downregulated in female offspring. Male and female F2 offspring displayed increased time spent in the open arm of the elevated-plus maze, suggesting lower levels of anxiety compared with control animals. Only male F2 offspring showed increased immobility time on the forced-swim test and increased latency to feed on the novelty-supressed feeding test, suggesting a depression-like phenotype in these animals. Collectively, these data provide evidence that paternal CORT treatment alters anxiety and depression-related behaviors across multiple generations. Analysis of the small RNA profile in sperm from CORT-treated males revealed marked effects on the expression of small noncoding RNAs. Sperm from CORT-treated males contained elevated levels of three microRNAs, miR-98, miR-144 and miR-190b, which are predicted to interact with multiple growth factors, including *Igf2* and *Bdnf*. Sustained elevation of glucocorticoids is therefore involved in the transmission of paternal stress-induced traits across generations in a process involving small noncoding RNA signals transmitted by the male germline.

## Introduction

There is now growing preclinical and epidemiological evidence supporting a transgenerational influence of paternal stress on the behavior of offspring in a manner not involving direct parenting interactions. Epidemiological studies of parents suffering from post-traumatic stress disorder (PTSD) have found a correlation with an increased instance of PTSD-like symptoms in the offspring, without the offspring being exposed to the trauma themselves.^1, 2, 3, 4^ Similarly, in rodents, early-life stress in the father can cause the first-generation offspring of male pups to display pro-depressive and heightened anxiety-related behavioral phenotypes, associated with altered expression of small RNA in the sperm of the father.^5^ This suggests that the paternal germline is susceptible to stress throughout life, potentially leading to transgenerational behavioral changes.

Stress in adult male mice can also lead to similar anxiety-like phenotypes in offspring to those seen in male mice exposed to stress as infants.^6^ Using a model of chronic social-defeat stress, Dietz *et al.*^6^ observed that the offspring of stressed adult male mice displayed increased depressive-like and anxiety-related behaviors. A common molecular pathology underlying stress-inducing transgenerational modification of the offspring behavioral phenotypes has not, however, been identified.

One commonality to the aforementioned models of traumatic stress is their impact on stress regulation, primarily through modification of the hypothalamic–pituitary–adrenal (HPA) axis activity. Increased levels of circulating corticosteroids are closely linked with anxiety and depression in both humans and rodents.^7, 8, 9^ Rodents chronically treated with oral corticosterone (CORT) develop suppression of HPA-axis activity and display depression-like behaviors and impaired fear extinction.^10, 11, 12, 13^ In addition, young and adult male mice subjected to a chronic variable stress paradigm sired offspring with blunted HPA-axis responses.^14^

The present study aimed to test whether dysregulation of the HPA axis using CORT administration results in a transgenerational effect of paternal stress. To do this, we treated adult male C57Bl/6 mice with CORT and then performed behavioral characterization of F1 and F2 offspring, focussing on parameters related to anxiety and depression. In an effort to define the molecular mechanisms underlying the observed transgenerational transmission of altered behavior, we analyzed the expression of genes related to stress, anxiety and depression. For example, underlying the behavioral impact of stress is a multitude of molecular changes that vary according to the approach, duration and timing of the stressors. Glucocorticoid receptors (GRs, *Nr3c1* gene) and mineralocorticoid receptors (MRs, *Nr3c2* gene) are the primary binding sites for circulating CORT. GRs act as the functional end point of the HPA axis, and abnormal expression levels have been associated with anxiety, depression and PTSD.^15, 16, 17^ Stress is known to decrease the levels of brain-derived neurotrophic factor (BDNF) expression in the hippocampus and has been strongly linked to depression and anxiety in both humans and rodent models.^18^ Of the multiple exons that undergo alternative splicing to create exon-specific transcripts of *Bdnf,*^19, 20^ changes to the expression of *BDNF* exon I and IV transcripts are associated with anxiety disorders in humans^21, 22^ and *Bdnf* exon IV is required for the extinction of conditioned fear in rodents.^23, 24^

Another gene potentially involved in paternal transgenerational effects is the paternally imprinted insulin-like growth factor II (IGF2). *Igf2* is required throughout development, and only the paternal copy is expressed.^25^ *Igf2* in the hippocampus regulates memory, dendritic plasticity^26^ and cellular turnover, which are deficient in anxiety disorders.^27^ In rodents, prenatal maternal stress has been shown to alter methylation of imprinted genes including *Igf2* (ref. 28), and knockout of placental *Igf2* has been shown to cause increases in anxiety-like behavior in the adult animal.^29^ A rodent study on the mechanism behind extinction learning has implicated *Igf2* hippocampal expression as being required for fear extinction.^30^ Blocking *Igf2* binding in the hippocampus impaired fear extinction, and increases in the level of *Igf2* were shown to facilitate extinction.^30^ Therefore, *Igf2* and *Bdnf* are likely to be relevant targets of stress and HPA-axis dysregulation.

We show that CORT treatment does not lead to behavioral changes in male breeders (F0). It does, however, induce transgenerational anxiety-related behavioral modifications in F1 and F2 offspring in a sex-specific manner. Altered behaviors could be detected from an early age (PND3), and were associated with changes in the hippocampal expression of *Bdnf* exon IV and *Igf2* mRNA in the F1 offspring. These changes are associated with the expression of regulatory RNAs contained in the sperm of CORT-treated male mice.

## Materials and methods

### Mice

Eight-week-old male C57BL/6 mice were purchased from the Animal Resources Centre (Murdoch, WA, Australia) and housed in the core animal facility in standard open-top laboratory mouse cages lined with sawdust and two tissues provided for nesting (15x30x12cm) with food and water *ad libitum.* Temperature and humidity were controlled at 22 °C and 45%, respectively. Mice were maintained on a 12-h light/dark cycle (lights on at 0700 hours) and bedding changed weekly. At 10 weeks of age, male mice (designated F0, paternal CORT) received either CORT-supplemented water (25 μg ml^−1^; Sterloids, Newport, RI, USA) or untreated water. After 4 weeks of CORT treatment, male mice were paired with naive 10-week-old C57Bl/6 females. Mice were pair-housed for up to 4 days and checked for vaginal plugs each morning. During this time, all mice received untreated water. Upon observation of a vaginal plug or after 4 days, female mice were separated and male mice resumed CORT treatment for 1 week before behavioral testing. Female mice were single-housed until they littered. F1 offspring were weaned at 4 weeks of age into cages of three to four mice per cage. Behavioral testing of offspring commenced at 8 weeks of age. F2 offspring were bred using 10–12-week-old F1 male mice (paternal CORT and untreated control) and 10–12-week-old naive females. All experiments were approved and performed in accordance with the guidelines of the Florey Institute of Neuroscience and Mental Health Animal Ethics Committee.

### Behavioral testing

All behavioral testing processes were performed during the light phase of the light/dark cycle, completing before 1200 hours when possible. Mice were acclimatized to the room for at least 1 h before commencement of each test, except for fear conditioning. Each test was performed on a separate day. See Supplementary Figure S1 for diagram illustrating test timing.

#### Maternal behavior

Maternal behavior toward the pups was observed daily from postnatal day (PND) 0–6 and scored as described previously.^31^ For more information see Supplementary Methods.

#### Ultrasonic vocalizations

Ultrasonic vocalizations (USVs) were tested on PND3. Each pup was removed from the litter and placed in a sound-recording chamber for 5 min where USVs were recorded using a microphone (Avisoft UltraSoundGate CM16 condenser ultrasound microphone, Avisoft Bioacoustics, Glienicke, Germany). USV analysis was performed using the Avisoft Sound Analysis and Synthesis Software pro (Avisoft Bioacoustics) using the automatic detection settings, with each call manually confirmed in accordance with previous studies.^32^ The number of USV calls made per minute over the 5-min separation period was determined. Where a call could not be differentiated from background noise, the animal was excluded from analysis.

#### Elevated-plus maze

Elevated-plus maze performance was tested at 14 weeks of age in the F0s and at 8 weeks in the offspring. The maze was made of light-colored Perspex with two open arms (5 × 30cm) and two enclosed arms (5 × 30 × 14 cm) extending from a central platform (5 × 5cm). Mice were placed in the center of the maze and monitored over a 5-min period. The testing room had subdued light levels (175 lux), which facilitated the automated tracking of the test subject using the TopScan tracking software (CleverSys, Reston, VA, USA). Total time spent in the open arms of the maze was expressed as a percentage of the test duration. Mice were excluded from analysis if they jumped off the maze (one control F2 male, one CORT F2 male and one control F2 female were excluded).

#### Light–dark test apparatus

Light–dark test apparatus performance was tested at 14 weeks of age in the F0s and at 8 weeks in the offspring. The light–dark test consisted of an open-field arena (ENV-510, Med Associates, Fairfax, VT, USA) was fitted with a dark box insert (ENV-511, Med Associates). The light half had a luminance of 700 lux. Mice started each 10-min test session in the dark half. Time spent in each half was automatically tabulated using the activity monitor software (Med Associates). Total time spent in the light half of the apparatus was expressed as a percentage of the test duration.

#### Forced-swim test

Forced-swim test was performed at 14 weeks of age in the F0s and at 8 weeks in the offspring and was used as a measure of depression-related behavior as previously described.^33^ Briefly, 2.5-l Perspex beakers were filled with water (23–25 °C), and mice were placed inside for 5 min. Each test session was video-recorded. Total immobility time over the last 240 s was manually scored by an experienced experimenter, and independently verified by a second experimenter. For the F2 cohort, the Depression Scan software (CleverSys) was used to automatically score the immobility time where the data were verified against manual scoring using regression analysis, and where scores lay outside 99% confidence interval that mouse was excluded. Three female mice from the control group and two male ones from the CORT group were excluded.

#### Novelty-suppressed feeding test

Novelty-suppressed feeding test (hyponeophagia) is a conflict-based test in which a food-deprived animal is presented a choice of approaching and consuming a food pallet in the center of a brightly lit, novel open arena or staying to the side and avoiding the center of the anxiogenic environment.^34^ Briefly, 12-week-old mice from a separate cohort were food-deprived for 48 h before testing but allowed 2 h of feeding after an initial 24-h period. Water was available *ad libitum*. Body weights pre- and post-deprivation were recorded. Mice lost ~15% body weight with no difference between groups (data not shown). Lighting was 350 Lux in the middle of test arena (80 × 80 × 80 cm), which was lined with 2 cm of bedding material, and a single food pellet was placed on a piece of filter paper in the center. Individual mice were placed into a random corner of the test arena, and the latency to grasp and feed on the food pellet within a 5-min timeframe was recorded.

#### Fear conditioning

Fear extinction is a learned behavioral response to stress of particular significance because it forms the basis of exposure therapies that are commonly used to treat anxiety disorders.^35, 36^ The initial fear-conditioning process is proposed to underlie the triggering of increased anxiety in PTSD. This may be due to a predisposition in these individuals to form abnormally strong or inappropriate links between stimuli, and experience of stress may underlie PTSD.^37, 38, 39, 40, 41, 42^ How fear memory is encoded has been shown to differ in juvenile animals compared with adult animals.^43, 44, 45^ Juvenile animals exposed to early-life stress display renewal of encoded fear memories similar to those seen in adult rodents.^45, 46^ Separate groups of behaviorally naive mice were subjected to fear-conditioning. Fourteen-week-old control and CORT-treated male mice, F1 offspring at PND15 ± 1 day and 8-week-old F1 offspring were tested. Two different contexts were created as described previously.^47^ During conditioning, all animals received 6 tone–foot-shock (CS-US) pairings (tone; volume: 80 dB; frequency: 5000 Hz, shock; 0.7 mA, 1 s).^47^ The day following conditioning, mice were tested for their conditioned stimulus (CS) memory (extinction) by being placed in a different context to that in which they were conditioned. Percentage freezing reported is based on 10 s of tone blocked into average freezing of 15 tones to represent early, middle and late extinction (see Supplementary Methods for more information).

### Physiological and molecular studies

Mice were killed by cervical dislocation for tissue collection between 0900 and 1200 hours. Blood was collected through cardiac puncture and left to clot at room temperature for 30 min. Following centrifugation at 1100 *g* for 15 min serum was collected and stored at −20 °C. Serum CORT levels were determined with an EIA Kit (Cayman Chemical, Ann Arbor, MI, USA) in triplicate according to the manufacturer's instructions. Post-stress samples were collected from mice immediately after forced-swim testing. Adrenal glands, testes, caudal epididymis and hippocampus were dissected, snap-frozen in liquid nitrogen and stored at −80 °C.

### Male fertility assessment

Male fertility was assessed using the strategy outlined previously.^48^ Briefly, following mating as outlined above, testes and epididymides were harvested from 14-week-old male mice. One testis was fixed in Bouin's fixative for histology and the second processed to calculate the daily sperm production.^49^ Sperm motility was assessed using a computer-assisted sperm analyzer.^50^ Sperm morphology was assessed at a light microscopic level following hematoxylin and eosin staining.

### Total RNA extraction and reverse transcription

Total RNA for real-time PCR studies was extracted using the QIAGEN RNeasy Mini extraction kit as per the manufacturer's instructions (QIAGEN, Chadstone, VIC, Australia). Tissue samples were disrupted using a Diagenode Biorupter (UCD-300; Liege, Belgium). On-column DNAse1 treatment was performed. RNA concentrations and purity were determined using Nanodrop spectrograph (2000c Thermo Scientific, Wilmington, DE, USA) and Agilent 2100 Bioanalyser Nanochips (Santa Clara, CA, USA). Overall, 1000 ng total RNA was reverse-transcribed using Superscript VILO cDNA Synthesis kit (#11754250 Invitrogen, Glen Waverley, VIC, Australia).

### Real-time quantitative PCR

Targeted gene expression was quantified with SYBR green PCR (S4438, Sigma-Aldrich, Castle Hill, NSW, Australia) using either the 7500 Fast Real-time PCR system sequence detection system (#4351107, Applied Biosystems, Carlsbad, CA, USA) or the Viia 7 Real-Time PCR system (#4453534, Applied Biosystems). For primer sequences and PCR conditions see Supplementary Methods.

### Small RNA extraction and Illumina sequencing

For small RNA-sequencing, mature spermatozoa were collected using the swim up method.^14^ For each treatment group, three pools each consisting of sperm collected from four individual mice were collected (that is, total 12 animals per treatment group). Samples were homogenized using QIAzol lysis reagent (QIAGEN). Quantification and quality control was performed on the total RNA using the Agilent Bioanalyzer 2100 with Small RNA kit (#5067-1548, Santa Clara, CA, USA); see Supplementary Figure S4 for representative electropherograms. Library preparation and sequencing was performed using the Illumina HiSeq2000 workflow (Australian Genome Research Facility). An amount of 500 ng of total RNA was used for library prep with 15 cycles of PCR. Quality control was performed following cDNA conversion using Agilent 2100 Bioanalyzer. All samples were run on a single flow cell lane using 50-bp single-end reads equating to an average of up to 20 million reads per sample; following removal of dimer and adapter sequences this was adjusted to between 2.1 and 7.6 M reads. Validation of the sequencing results was performed on sperm samples collected from five control and five CORT-treated mice.

### Sequencing analysis

Read quality was assessed using FastQC and were trimmed against known common Illumina adapter/primer sequences using trimmomatic.^51^ All reads were then aligned to mouse genome mm10 using the Subjunc aligner within the Subread package.^52^ Sequencing data were then summarized into Reads per transcript using Featurecounts^53^ and the Gencode M2 gene models for the mouse mm10 genome build (July 2013 freeze).^54^ Normalization and statistical analysis were executed using EdgeR^55, 56, 57^ (for more detailed information see Supplementary Methods).

For analysis of predicted targets, only the functionally described small RNAs were used. Initially, small RNA transcripts that were significantly increased by log2 fold-change of 2 or more were included. From that list, the top 20 significantly changed small RNAs were inputted into the miRWalk program.^58^ For more detailed information see Supplementary Methods.

### Statistical analysis

Student's *t*-tests (unpaired) were used to determine differences between two the means, where appropriate. Analyses of variance were used to examine main effects (for example, paternal CORT treatment) and/or interactions. Repeated measures analyses of variance were used to analyze data collected from multiple time points (for example, USVs and maternal observations). Bonferroni *post hoc* tests were used to determine specific differences in the event of significant interactions. Significance was set at *P*<0.05. Statistical analyses were performed using SPSS statistics version 22.0 (IBM, Armonk, NY, USA) and GraphPad prism 6.0 (GraphPad software, LA Jolla, CA, USA). All graphs presented as mean±s.e.m.

## Results

### CORT treatment alters F0 physiology with no changes to behavior

**Figure 1:**
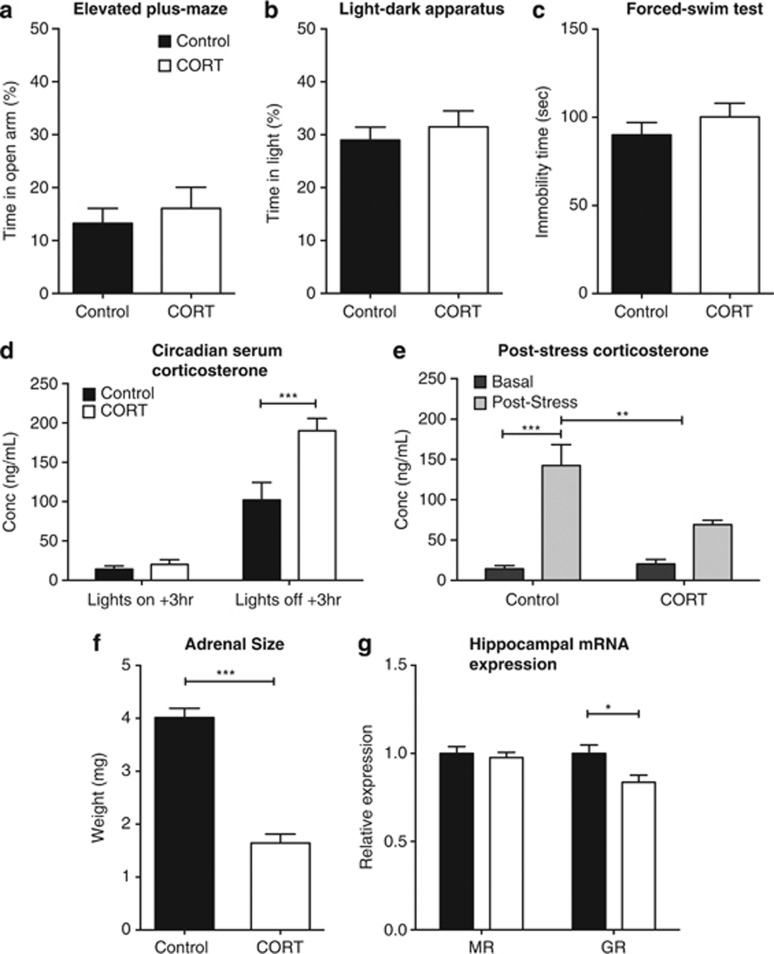
Effect of corticosterone (CORT) on F0 behavior and physiology. CORT administration did not affect F0 behavior on the elevated-plus maze (*n*=23–24) (**a**), the light–dark apparatus (*n*=12; **b**) or the forced-swim test (*n*=24; **c**). CORT administration significantly increased the level of serum CORT 3 h following the beginning of the active phase (*n*=4–5; **c**) and resulted in a significant decrease in the post-stress response of serum CORT (*n*=6; **d**). CORT caused a significant decrease in the adrenal weights of the F0 animals (*n*=8–10; **e**) and, although there were no changes to the hippocampal expression of mineralocorticoid receptor (MR), there was a significant reduction in glucocorticoid receptor (GR) mRNA (**f**). *P<0.05, **P<0.01, ***P<0.001, values represent means±s.e.m.

Four weeks of CORT treatment did not influence anxiety or depression-related behavioral measures of male F0 mice. There was no significant difference in the time spent in the open arms on the elevated-plus maze (*t*_(45)_=0.5874, *P*=0.5874; Figure 1a) nor on the total distance traveled (*t*_(45)_=0.3547, *P*=0.7244). Similarly, no changes were observed in the time spent in the light half of the light–dark apparatus (*t*_(29)_=0.6409, *P*=0.5266; Figure 1b) or the total distance traveled (*t*_(29)_=1.326, *P*=0.1950). There was no effect of CORT treatment on total immobility time during the forced-swim test (*t*_(46)_=0.98, *P*=0.3322; Figure 1c).

In order to demonstrate a significant increase in serum CORT levels associated with CORT treatment, serum CORT levels were quantified at 3 h after lights on and 3 h after lights off (the latter corresponding to peak fluid consumption). There were significant effects of collection time (F_(1,14)_=103.61, *P*<0.0001) and CORT treatment (F_(1,14)_=13.89, *P*=0.0023), as well as a time × treatment interaction (F_(1,14)_=10.55, *P*=0.0058; Figure 1d). *Post hoc* tests revealed a significant difference between CORT and control animals 3 h after lights off (*P*<0.001).

CORT treatment was also a significant modifying factor of serum CORT levels post-forced-swim stress (F_(1,18)_=5.090, *P*=0.0368). There was also an overall effect of stress (F_(1,18)_=35.14, *P*<0.0001) and a significant CORT × stress interaction (F_(1,18)_=7.08, *P*=0.0159; Figure 1f). *Post hoc* analysis showed that serum CORT levels were significantly increased in untreated control mice post stress (*P*<0.0001). Serum CORT levels of untreated mice post stress were also significantly greater than those of CORT-treated mice (*P*<0.01; Figure 1e). CORT treatment significantly reduced adrenal wet weight compared with controls (*t*_(16)=_9.546, *P*<0.001; Figure 1f).

We examined GR and MR expression in the F0 hippocampus as both are key regulators of HPA-axis activity, besides being linked to anxiety phenotypes.^15, 16, 17^ CORT treatment reduced hippocampal GR mRNA levels (*t*_(18)_=2.793, *P*=0.012); however, there was no change in MR expression (*t*_(18)_=0.51697, *P*=0.6097; Figure 1g). There were no significant effects of CORT treatment of GR and MR expression in cortical tissue (data not shown).

Male fertility was not affected in CORT-treated males as indicated by comparable mating frequency and litter sizes to those seen in control-treated males (see Supplementary Figure S2). The average number of female and male pups born per litter did not differ significantly (see Supplementary Figure S2). Similarly, daily testis sperm production, testis weight and sperm motility were unaffected (Supplementary Fig. S3). Dams that mated with CORT-treated males did not display different levels of maternal licking and grooming behavior toward pups (see Supplementary Figure S2).

### Altered F1 female offspring phenotypes associated with paternal CORT treatment

**Figure 2:**
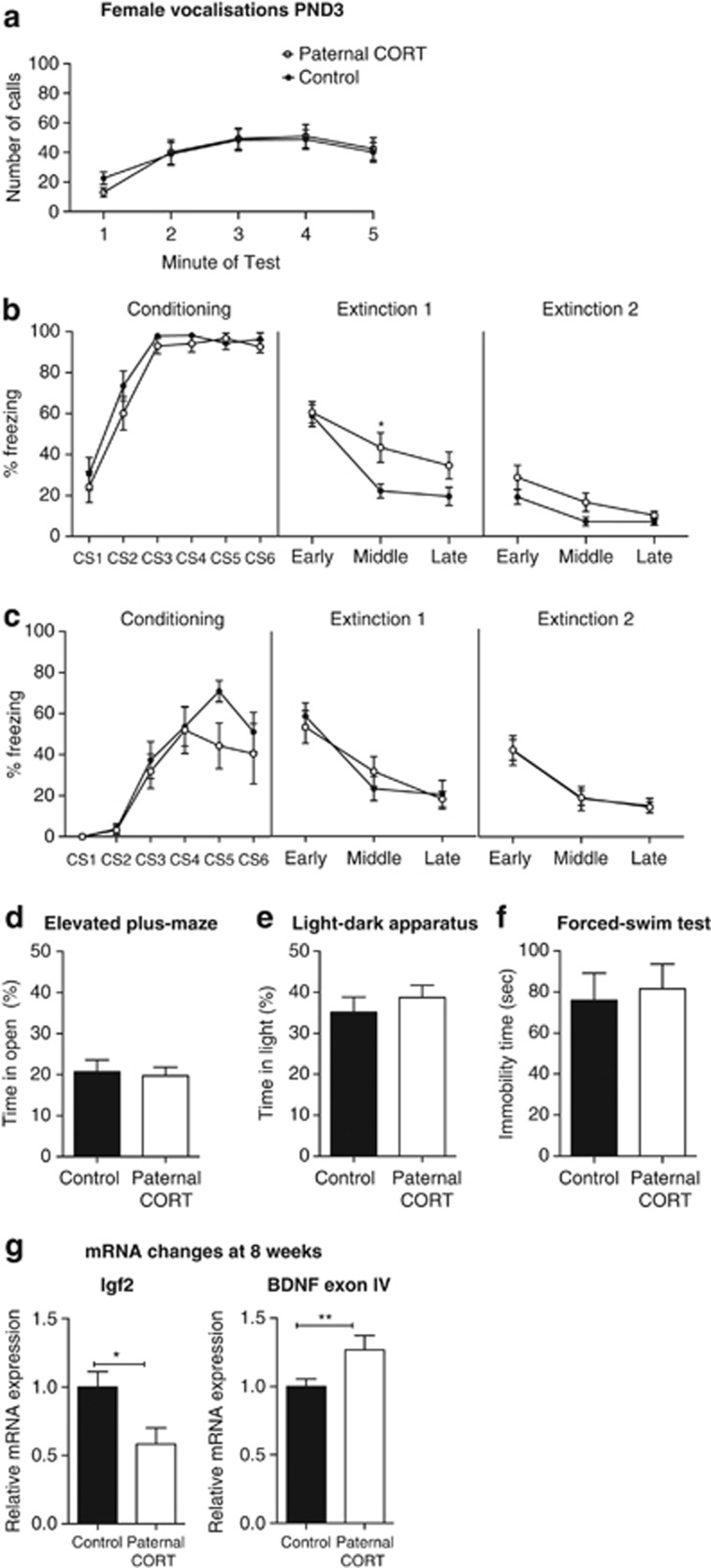
Paternal CORT treatment changes early-life fear conditioning, with no changes to behavior in adulthood in female offspring. Paternal CORT has no effect on the number of vocalizations made by female animals (**a**). At 2 weeks of age, the mean levels of freezing to the conditioned stimulus (CS) during the conditioning trial, and extinction trial day 1 and day 2, respectively *n*=22–25 (**b**) per group. At 8 weeks of age, there were no differences in the mean levels of freezing to the CS during the conditioning trial and extinction trial days 1 and 2, respectively (**c**) *n*=8–12 per group. As adult offspring, paternal CORT treatment had no effect on the time spent in the open of the elevated-plus maze (**d**; *n*=23 per group), or the time spent on the light side of the light–dark apparatus (*n*=12–14 per group; **e**). Paternal CORT had no effect on the total time immobile in the forced-swim test (*n*=23–14 per group; **f**). At 2 weeks of age, there were no changes in the expression of *Igf2* mRNA in the hippocampus; however, there was a significant decrease in the level of hippocampal *Bdnf* exon IV mRNA (**g**; *n*=4 per group). At 8 weeks of age, *Igf2* mRNA was significantly decreased in the hippocampus of F1 females born to CORT fathers (*n*=6–7 per group), whereas there was a significant increase in the level of *Bdnf* exon IV (*n*=8 per group; **h**). *P<0.05, **P<0.001, values represent means±s.e.m. CORT, corticosterone.

As previous studies have found sex differences in the offspring of stressed fathers, all analyses performed on the offspring were conducted on males and females independently.^5, 6, 14, 59^ PND3 female pups made increasing numbers of USVs over the 5-min separation period (F_(1,136)_=10.252, *P*<0.0001); however, there was no difference attributable to paternal CORT treatment (F_(1,34)_=0.009, *P*=0.927) and no time × paternal CORT interaction (F_(4,136)_=0.391, *P*=0.815; Figure 2a).

When PND15 female pups underwent fear conditioning (Figure 2b), there was an overall effect of CS on the conditioning day (F_(5,225_)=63.286, *P*<0.0001) but no overall effect of paternal CORT (F_(1,45_)=2.178, *P*=0.147) nor a CS × paternal CORT interaction (F_(5,225_)=0.5254, *P*=0.757), suggesting that both groups conditioned equally to the tone. On the first extinction day there was a trend for an effect of paternal CORT treatment on freezing (F_(1,45)_=3.728, *P*=0.06), with a significant extinction × paternal CORT interaction (F_(2,90)_=3.201, *P*=0.045). *Post hoc* analysis revealed that there was a significantly higher level of freezing behavior displayed by the female offspring of paternal CORT-treated mice during the middle blocks of extinction (*t*_(135)_=2.658, *P*=0.0262). On the second extinction day there was a similar trend for an effect of paternal CORT treatment (F_(1,45)_=3.188, *P*=0.057). However, the extinction × paternal CORT interaction was not significant (F_(2,90)_=0.656, *P*=0.521).

A separate cohort of 8-week-old animals was used to assess adult fear conditioning. Females at 8 weeks of age showed a significant effect of CS exposure (F_(5,90)_=19.338, *P*<0.0001) but no effect of paternal CORT (F_(1,18)_=2.164, *P*=159) and no CS × paternal CORT interaction (F_(5,90)_=1.664, *P*=0.213) on the female offspring at 8 weeks of age. Therefore, both groups conditioned equally to the tone over the trial. On the first extinction day there was no overall effect of paternal CORT (F_(1,18)_=0.001, *P*=0.970), and no significant interaction (F_(2,36)_=1.452, *P*=0.247). On the second day of extinction there were no effects of paternal CORT (F_(1,18)_>0.0001, *P*<0.999) nor any significant interaction (F_(2.36)_=0.011, *P*=0.989).

There were no observed differences in the average time spent in the open arms of the elevated-plus maze (*t*_(44)_=0.2666, *P*=0.7910; Figure 2d) or the total distance traveled in the plus maze (*t*_(44)_=0.4497, *P*=0.6552) and time spent in the light half of the light–dark apparatus (*t*_(24)_=0.7427, *P*=0.4649) or the total distance traveled (*t*_(24)_=0.7145, *P*=0.4818; Figure 2e). No differences were observed in the forced-swim test (*t*_(45)_=0.3120, *P*=0.7565; Figure 2f).

### Changes to hippocampal gene expression associated with paternal CORT treatment in female offspring

As in the F0 animals, we examined GR and MR expression in offspring hippocampi. In addition, we analyzed the expression of *Bdnf* and *Igf2* because of their regulatory roles in anxiety, depression and the extinction of fear memory.^18, 19, 20, 21, 22, 23, 24^ There were no overall effects of paternal CORT treatment on GR or MR mRNA levels (data not shown).

At 8 weeks of age, however, there was a significant decrease in the expression of hippocampal *Igf2* mRNA (*t*_(11)_=2.646, *P*=0.0228). Whereas there were no significant changes in the expression of total *Bdnf* in the hippocampus (data not shown), *Bdnf* exon IV transcript levels were significantly greater in the hippocampus of female paternal CORT offspring (*t*_(14)_=2.424, *P*=0.0295; Figure 2h). Collectively, these data indicate that paternal CORT can significantly alter anxiety-associated gene expression in the brain of the female offspring and is associated with variable changes in behavior.

### Altered F1 male offspring phenotypes associated with paternal CORT treatment

**Figure 3:**
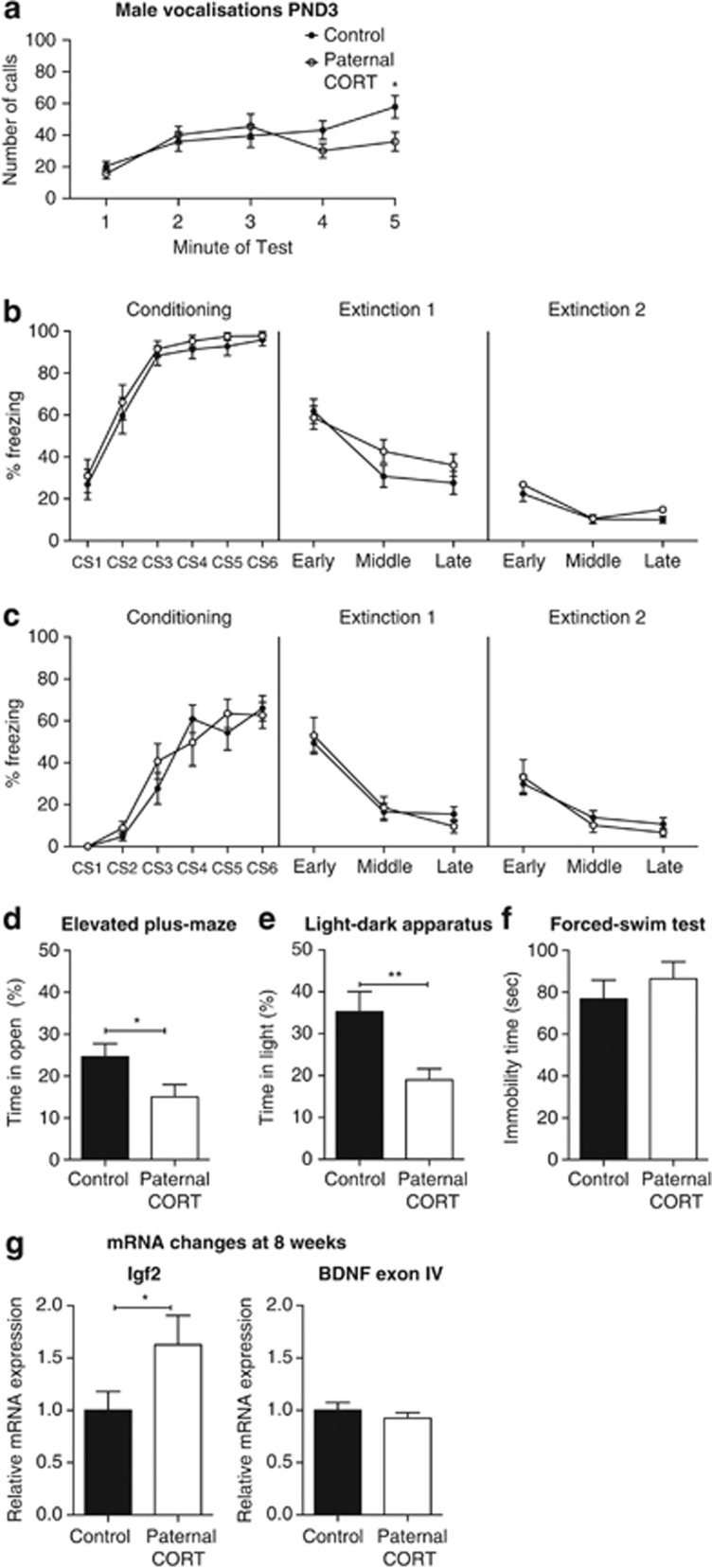
Paternal CORT treatment is associated with altered anxiety-related behaviors of male offspring. Male offspring of CORT-treated mice made fewer calls during the last minute of separation (**a**; *n*=19–21 per group). Both 2-week (**b**; *n*=24 per group) and 8-week-old (**c**; *n*=15 per group) male F1 offspring display no differences in fear conditioning and extinction trials. Male offspring of CORT-treated mice spent less time in the open arms of the elevated plus-maze (EPM) (**d**; *n*=19 per group) and less time in the light half of the light–dark box (**e**; *n*=12 per group). No difference was observed on the forced swim test (FST) (**f**; *n*=23–24 per group). There was a significant increase in the level of *Igf2* mRNA expression in the hippocampus; however, there were no changes in the expression of *Bdnf* exon IV expression in the hippocampus (**g**) *n*=8. *P<0.05, **P<0.001, values represent means±s.e.m. CORT, corticosterone.

At PND3, male F1 offspring from CORT-treated male mice made greater number of USVs over time compared with offspring from control male mice (F_(4,144)_=10.594, *P*<0.002). There was no overall effect of paternal CORT treatment (F_(1,36)_=1.093, *P*=0.303); however, there was a significant time × paternal CORT interaction (F_(4,144)_=2.969, *P*=0.022).* Post hoc* analysis revealed that the pups born to paternal CORT-treated mice made significantly fewer calls in the final minute of the test (*t*_(180)_=2.6329, *P*=0.0452; Figure 3a).

When tested for fear conditioning at 2 weeks of age, there was a significant main effect of CS on conditioning day (F_(5,230)_=50.55, *P*<0.0001) but no main effect of paternal CORT (F_(1,46)_=0.922, *P*=0.342) nor a significant CS × paternal CORT interaction (F_(5,230)_=0.47, *P*=0.999). Therefore, both groups conditioned equally to the CS. On the first extinction day, there was no effect of treatment (F_(1,46)_=0.766, *P*=0.386) or extinction × paternal CORT interaction (F_(2,92)_=2.538, *P*=0.085). Similarly, there was no main effect of paternal CORT (F_(1,46)_=0.816, *P*=0.371) nor extinction × paternal CORT interaction (F_(2,92)_=0.349, *P*=0.707) on second extinction day (Figure 3b). Unlike the female mice, the male offspring of paternal CORT male mice showed no impairment in extinction learning.

A separate group of male mice underwent fear conditioning at 8 weeks of age. During the conditioning trial, there was a significant effect of CS presentation (F_(5,100)_=53.19, *P*<0.0001) and no effect of paternal CORT (F_(1,20)_=0.11, *P*=0.7417) or interaction (F_(5,100)_=1.33, *P*=0.2586) as both groups equally conditioned to the tone. During the extinction trials, there were no effects of treatment (F_(1,20)_<0.0002, *P*=0.9871) or a significant interaction (F_(2,40)_=1.08, *P*=0.3484) on either the first day or the second day (F_(1,20)_=0.09, *P*=0.7728; F_(1,40)_=0.75, *P*=0.4768; Figure 3c).

When tested for anxiety and depression-like phenotypes as adults, the male offspring of paternal CORT-treated mice spent significantly less time in the open arms of the elevated-plus maze (*t*_(36)_=2.241, *P*=0.0313; Figure 3d), with no change to the total distance traveled (*t*_(36)_=0.7208, *P*=0.4757), and significantly less time in the light half of the light–dark apparatus (*t*_(22)_=2.973, *P*=0.0070; Figure 3e), with no change to the distance traveled (*t*_(22)_=1.762, *P*=0.0920). There was no difference in average immobility time during the forced swim test (*t*_(45)_=0.7884, *P*=0.4346; Figure 3f). These data indicate significant alterations to the anxiety-associated behaviors in the male offspring of CORT-treated fathers.

### Changes to hippocampal gene expression associated with paternal CORT treatment in male offspring

As in the female offspring, paternal CORT treatment did not affect expression of GR or MR mRNA expression in male offspring.

In male offspring of CORT fathers there was, however, a significant increase in the level of *Igf2* mRNA expression in the hippocampus at 8 weeks of age (*t*_(14)_=2.2, *P*=0.0451; Figure 3g). As in the females, there were no effects on total *Bdnf* or *Bdnf* exon I mRNA levels in the hippocampus of paternal CORT male offspring. However, there were also no effects of paternal CORT on *Bdnf* exon IV expression in the hippocampus (Figure 3g; *t*_(14)_=0.928, *P*=0.3694).

### Paternal CORT treatment exerts selective behavioral effects on the F2 generation

**Figure 4:**
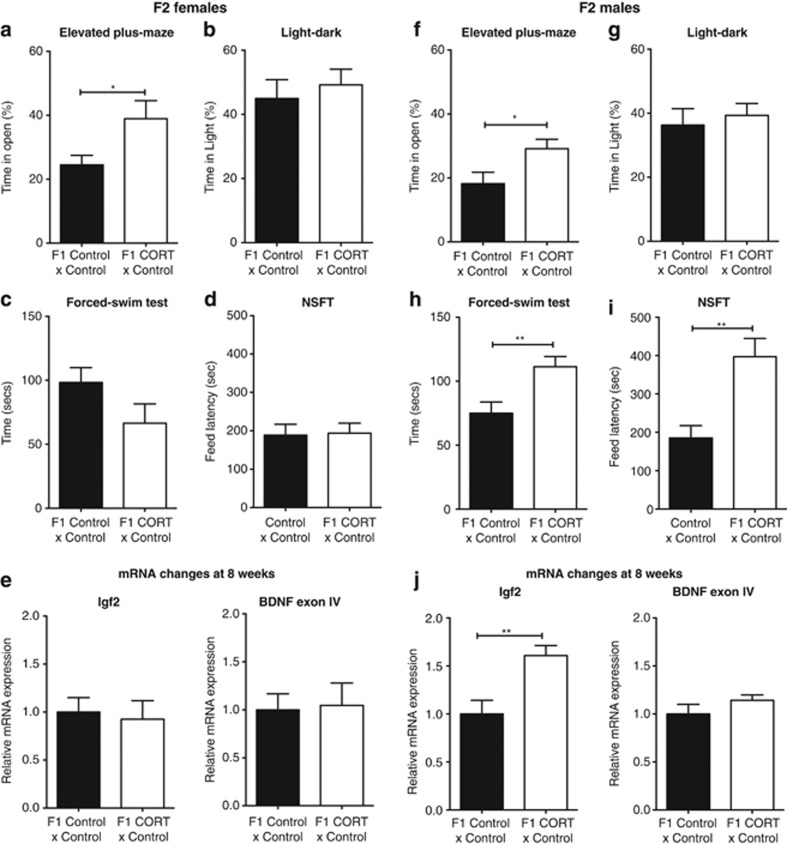
Transgenerational effects of paternal CORT treatment on F2 anxiety and depression-related behaviors. F2 female mice born to F1 CORT male mice exhibited greater time in the open arms of the elevated-plus maze (*n*=7–13 per group; **a**), but no difference was observed in the light–dark box (*n*=9–14 per group; **b**). There was no significant difference in depression-related behaviors as assessed with the forced-swim (*n*=10–11 per group;** c**) and novelty-suppressed feeding (*n*=7–13 per group; **d**) tests. There were no significant changes to hippocampal mRNA expression of *Igf2* or *Bdnf* exon 4 (*n*=8 per group; **e**). F2 male mice born to F1 CORT male mice also exhibited more time in the open arms of the elevated-plus maze (EPM) (*n*=13–14; **f**) but with no difference observed in the light–dark box (*n*=12–14 per group) (**g**). However, F2 CORT male mice exhibited pro-depressive behaviors in the forced-swim (*n*=12–17 per group;** h**) and novelty-suppressed feeding (*n*=6–10 per group; **i**) tests. There was a significant increase in the level of *Igf2* mRNA expression in the hippocampus of F2 males; however, there were no changes in the expression of *Bdnf* exon IV expression in the hippocampus (*n*=7–8 per group; **j**). Data represented as mean±s.e.m. Unpaired *t*-test: *P<0.05, **P<0.01. CORT, corticosterone.

In order to determine whether paternal CORT treatment exerted transgenerational effects on behavioral phenotypes beyond the F1 generation, we mated F1 male mice from both treatment arms with F1 control female mice. F2 female mice sired by F1 CORT male mice recorded significantly greater time in the open arms of the elevated-plus maze (Figure 4a; *t*_(18)_=2.518, *P*=0.0215). However, no difference was observed in the light–dark box (Figure 4b; *t*_(21)_=0.5067, *P*=0.6176). There were also no differences in immobility time in the forced-swim test (Figure 4c; *t*_(19)_=1.692, *P*=0.1069) and latency to feed in the novelty-suppressed feeding test (Figure 4d; *t*_(18)_=0.135, *P*=0.894). No changes were observed in hippocampal mRNA expression of *Igf2* (*t*_(14)_=0.04135, *P*=0.9676) or *Bdnf* (*t*_(14)_=0.7208, *P*=0.4829) in the female F2 generation (Figure 4e).

Similarly, F2 male mice sired by F1 CORT male mice spent significantly more time on the open arms of the elevated-plus maze (Figure 4f; *t*_(25)_=2.344, *P*=0.0274); however, no difference was detected in the light–dark box (Figure 4g; *t*_(24)_=0.4898, 0.6287). In contrast to the female mice, F2 male mice bred from F1 CORT male mice were observed to spend greater time immobile during the forced-swim test (Figure 4h; *t*_(27)_=2.911, *P*=0.0071), besides displaying greater latency to feed in the novelty-suppressed feeding test (Figure 4i; *t*_(14)_=3.169, *P*=0.007). Similarly to the male F1 generation, the male F2 generation showed a significant increase in hippocampal mRNA expression of *Igf2* (*t*_(13)_=3.692, *P*=0.0027); however, there was no change in the expression of *Bdnf* exon IV (*t*_(13)_=2.103, *P*=0.0555; Figure 4j).

### CORT treatment alters sperm microRNA population

Small RNA-sequencing was performed to determine whether CORT treatment alters sperm microRNA (miRNA) expression patterns, which have previously been linked to paternal transgenerational modification of offspring phenotypes.^14, 60^

**Figure 5:**
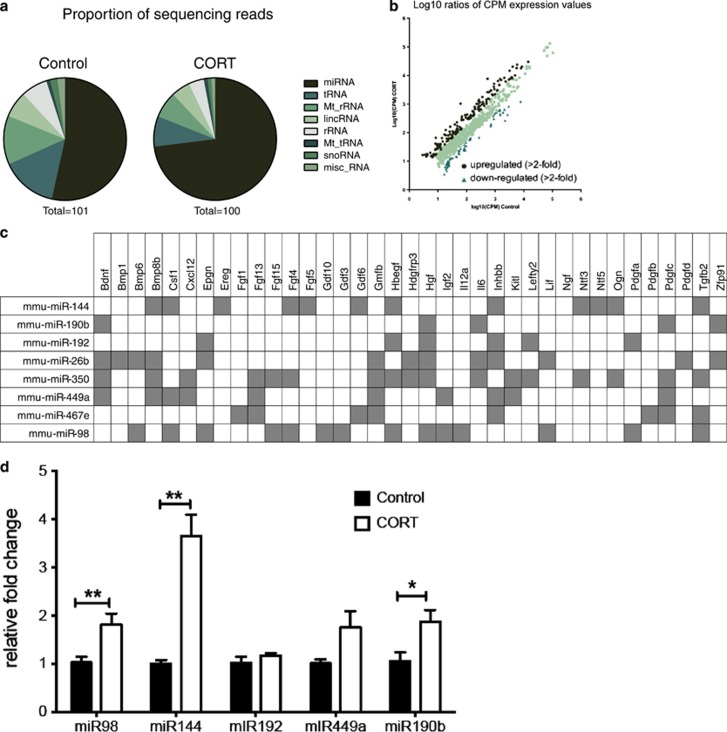
CORT treatment alters microRNAs (miRNAs) in sperm. Distribution of various types of small RNAs in the sperm of control and CORT-treated mice (**a**). Expression value of genes with altered expression compared with the average of the control animals (**b**). Genes that have predicted binding sites for miRNAs found to have altered expression in the sperm of CORT-treated animals (**c**). Fold change (relative to SNORD95) of top miRNA candidates from sequenced sperm (**d**). *P<0.05, **P<0.01, values represent means±s.e.m. CORT, corticosterone.

Our study found 188 differentially expressed small noncoding RNA sequences that mapped to the current mouse genome (false discovery rate-adjusted *P*<0.1; Figure 5d). Of these, the two largest proportions were miRNAs (101 transcripts, 54%) and tRNAs (61 transcripts, 32%). The remainder comprised 11 predicted small RNAs (Gm), 5 small nucleolar RNAs (Snord), 3 nuclear encoded rRNAs (5 S) and 3 mitochondrially encoded tRNAs (Figure 5a). Analysis of transcripts found that there were 166 with a fold-change counts per million greater than twofold that of the sperm of CORT-treated animals and 105 small RNAs, which were downregulated twofold compared with controls (Figure 5b). See Supplementary Table S1 for list of twofold upregulated genes. In order to investigate the mechanism underlying the observed transgenerational effects, we focused on miRNAs that were changed by twofold or greater, that is, a total of 46 miRs. The list was sorted in an ascending order of *P*-values, and the top 20 ranked miRNAs were applied to miRWalk, which returned a predicted 8200 downstream gene targets.^58^ The data were filtered further using the online bioinformatics tool, DAVID, to functionally annotate potential involved signaling pathways^61, 62^ (Figure 5c).

We observed that, within the functionally annotated pathways, growth factors featured prominently. We probed miRNAs predicted to regulate growth factors further since we had observed changes in two essential growth factors, *Igf2* and *Bdnf,* in the offspring hippocampus. Both genes have predicted binding sites for miRNAs that were shown to have increased expression in the CORT sperm. miR-190b, miR-26b, miR350 and miR-449a have predicted binding sites for *Bdnf*. miR-192, miR-449a and miR-98 have predicted binding to *Igf2* (Figure 5c).

An independent cohort of CORT-treated animals was generated to validate the five top miRNA candidates, miR-190b, miR-192, miR-449a, miR-98 and miR-144, using SNORD95 as a reference gene (Figure 5d). Three of these candidate miRNAs were validated as being more highly expressed in the sperm from CORT-treated mice, namely miR-98 (*t*_(6.4)=_5.26, *P*=0.002), miR-144 (*t*_(3.9)_=5.93, *P*=0.008) and miR-190b (*t*_(7.4)_=2.73, *P*=0.028). There was no significant change in the expression of miR-192 (*t*_(5.5)_=1.15, *P*=0.335). Expression of miR-449a was also not significantly altered by CORT treatment (*t*_(4.4)_=2.16, *P*=0.091), although variability in the CORT-treated samples was significantly different from controls (*P*=0.014; Figure 5d).

## Discussion

We believe we show for the first time that the direct manipulation of CORT levels as a model of chronic physiological stress exerts a transgenerational influence on offspring behaviors and gene expression via the male germline. Our results agree with, and substantially extend, previous studies that have investigated mouse models of early-life and traumatic stress.^14, 60^ The present study supports and strengthens the evidence of paternally driven transgenerational modification of offspring phenotypes. The use of CORT supplementation firmly implicates the dysregulation of glucocorticoid signaling and its consequential modification of sperm miRNA content as the major physiological events facilitating the transgenerational influence.

Male offspring were more affected by paternal CORT treatment through both F1 and F2 generations. Male, but not female, F1 offspring had reduced vocalizations during PND3 maternal separation (previously suggested to be indicative of an early dysregulation of the HPA axis^63^). This phenotype at an early age was consistent with the pro-anxiety behaviors observed in adult F1 males that were, again, not detected in female F1s. Furthermore, male F1 offspring were susceptible to the effects of paternal stress in the absence of additional negative genetic or environmental triggers for developing pro-anxiety behaviors.

Male F2 offspring also developed additional pro-depressive characteristics by adulthood (increased immobility in forced swim test and increased latency to feed) that were not observed in female F2 offspring. Thus, behavioral modifications across multiple generations can be elicited via the male germline, albeit with different directionality of the phenotypic changes. The existence of transgenerational influence of paternal stress on offspring-affective phenotypes remains to be verified in humans, but it certainly poses an intriguing scenario of potentially major significance to public health. It is imperative that such a possibility is explored through clinical studies.

This is the first study to examine the effect of paternal stress on fear conditioning in offspring at an early age. Fear extinction is a learned behavioral response to stress of particular significance because it forms the basis of exposure therapies that are commonly used to treat anxiety disorders.^35, 36^ The initial fear-conditioning process is proposed to underlie the triggering of increased anxiety in PTSD. This may be because of a predisposition in individuals to form abnormally strong or inappropriate links between stimuli, which may underlie PTSD following stressful experiences.^37, 38, 39, 40, 41, 42^ This study has revealed a deficit during the initial extinction phase in the female offspring of CORT-treated fathers. This is consistent with a previous report in adult mice showing that females take longer to extinguish learned fear compared with males.^23^ However, despite the initial extinction impairment, this appears to be a subtle deficit as female offspring ultimately achieved similar levels of extinction following further exposures to the CS. Interestingly, the F1 females did not develop any behavioral changes after sexual maturation. The absence of similar behavioral modifications in the female offspring compared with the male offspring, however, does not imply that females are resistant to the transgenerational effects of paternal stress. Rather, it may be that the female F1 offspring of CORT-treated mice require an additional stressor or negative life event to display phenotypes. It remains to be determined whether exposure to a subthreshold, low-level stressor could be sufficient to elicit pro-anxiety or pro-depressive behaviors in offspring of CORT-treated fathers.

Our data add to the growing evidence of transgenerational effects on offspring phenotypes, which manifests differently in males and females. The seminal epidemiological studies on the nutrition of grandparents exerting transgenerational influence on health outcomes revealed sex-specific effects depending on the grandparent in question.^64, 65^ Similar to our male germline-linked observations, when the paternal grandfather was born in a time of good food supply, their grandsons (that is, F2 generation) but not grand-daughters had greater mortality risk ratio.^64, 65^ In contrast, the paternal grandmothers' food supply only influenced the mortality of their grand-daughters and not grandsons, with no effect of the maternal grandparents for the children of either sex.^64^ In addition, paternal smoking starting at an early age has been linked to increased body mass index of their male children, but not the females (even when adjusted for continued smoking up to conception).^64^ These findings point to a male germline-mediated influence over certain offspring physical traits, which is dependent on paternal lifestyle factors. Thus, there is strong impetus to conduct human studies to establish the transgenerational effects of paternal stress.

Previous mouse transgenerational studies of paternal stress have reported inconsistent results regarding the directionality of the sex-specific effects.^5, 6^ For example, early-life stress resulted in female F1 offspring with deficits in the free exploratory paradigm and open-field test, but no changes were observed in the male F1 offspring.^5^ In contrast, social defeat stress imposed on adult males resulted in F1 offspring (male and female) with behavioral impairments on the elevated-plus maze and forced-swim test; yet only the male offspring displayed additional behavior deficits in novelty exposure and anhedonia.^6^ Whereas both maternal separation and social defeat paradigms are well established, robust and reproducible behavioral paradigms and the collective evidence indicates that paternal stress is linked to increased offspring anxiety, the different outcomes in terms of the transgenerational effects clearly demonstrate disparate reproductive consequences of both approaches. Whereas each one is a good model of specific trauma-related conditions, it has been a challenge to interpret these findings in a manner that is relevant beyond clinical subpopulations.

Our study, through direct manipulation of CORT levels, was an attempt to address the contrasting transgenerational effects on offspring behavior caused by different stress interventions. In using this purely physiological approach, we are able to target the HPA axis during adulthood directly. However, despite establishing a clear behavioral impact on the F1 and F2 generations, our study might not reflect the full spectrum of paternal stress effects because physical (for example, restraint) and anxiogenic stress (for example, maternal separation) activate a complex interacting network of ethologically relevant systems,^66^ and elevates the levels of other neuroactive hormones including adrenalin, adrenocorticotropic hormone, corticotropin-releasing hormone and vasopressin.

Given prolonged exposure to stressful situations and increased levels of corticosteroids are closely linked with anxiety and depression in both humans and rodents,^7, 13, 67^ it was unexpected to not observe behavioral deficits in sires as a result of CORT treatment. However, the published protocol describing oral CORT administration as a rodent model of depression requires withdrawal of CORT in order to manifest the depression phenotype in the animals.^13^ There is one study describing a similar oral CORT administration paradigm, which performed depression tests (tail-suspension and forced-swim tests) without a withdrawal period.^67^ However, that study used a significantly longer administration period of 8 weeks, and the juvenile animals were treated from 3 weeks of age, making it more a model of persistent stress originating from early life. We were interested in the effects of CORT treatment during adulthood in order to be able to address the issue of germline transmission, independent of CORT-induced paternal behavioral changes. It is important to note that, whereas we established that CORT treatment did not result in a paternal behavioral difference according to our protocols, it is possible that behavioral differences could be present during the active phase when serum CORT levels were significantly elevated (that is, 3 h after lights off). Future studies could be designed to address this.

As separate research groups have independently demonstrated that paternal stress (be it behavioral interventions or physiological as the present study) experienced before mating is linked to modified offspring behaviors and physiology, the present research focus now shifts to the elucidation of the molecular mechanisms that underpin offspring phenotypes. There is evidence that miRNAs are likely to have a major role.^14, 60, 68^ Our RNA-sequencing data support, and substantially extend, these findings, with CORT treatment associated with a shift in the proportions of small RNAs contained in sperm as well as the levels of a subpopulation of miRNAs. We provide new insight into the propensity for noncoding RNA content of spermatozoa to be modified by non-genetic factors, demonstrating that stress hormone can modulate transgenerational epigenetics. Given the 28-day spermatogenic cycle of *Mus musculus* and the necessity for ~1 week of epididymal sperm maturation before the attainment of fertilization competency, the implications derived from the 4-week treatment duration used in this study is that any modification of small RNA content occurs in post-meiotic germ cells, either in the testis or in the epididymis. Transfer in the epididymis could, for example, be mediated via epididymosomes and is supported by studies suggesting miRNA transfer in wild-type mice under normal physiological conditions.^69, 70^ Further investigation of the role of the other subtypes of small noncoding RNAs (for example, tRNAs) in the transgenerational influence is also warranted. In addition, a role for DNA methylation should not be discounted. Previous work has shown changes in sperm DNA methylation following altered paternal diet,^5, 71, 72^ and paternal stress has been shown to change DNA methylation patterns in offspring.^73, 74^ It is possible that altered DNA methylation marks are passed on through the sperm or that changes to sperm small RNA content causes altered methylation in the offspring tissue.

Thus far, only four miRNAs have been commonly reported as being upregulated in the sperm of stressed fathers, namely miR-30c, miR-204, miR-375 and miR-532.^14, 60^ Although none of the four aforementioned miRNAs were altered in this present study, we did find some agreement with miR-30a and miR-32, both being present in the data set of Rodgers *et al*,*.*^14^ and miR-21a, miR-26b, miR-30b, miR-103, miR-194, miR-449a found to be upregulated in both the present study and in that of Gapp *et al*.^60^ In addition, let7f, let7g and let7i were found to be upregulated in both this study and Gapp *et al.*^60^ It is important to emphasize that we are not discounting the importance of other small RNAs not common between the studies to-date because they are potentially acting as secondary molecular mediators of the offspring phenotypes under specific study conditions.

MicroRNAs serve a crucial regulatory function during embryonic development,^75, 76, 77^ but how paternal stress via modification of sperm miRNA content translates to the molecular and phenotypic shifts observed in offspring generations remains to be determined. It is possible that miRNAs are able to modify behavior by either directly regulating specific neuronal genes within brain regions that modulate behavior (for example, hippocampus and amygdala), or through the regulation of transcription factors to bring about the gene expression differences, which in turn lead to modified behavioral outputs. Alternatively, it is possible that certain miRNAs regulate other epigenetic modifications such as DNA methylation or histone modifications, which would be sufficient to induce whole-of-life changes in gene expression and, consequentially, behavior. This latter scenario is yet to be investigated in the context of paternal transgenerational stress effects. There is yet to be mechanistic evidence that miRNA differences are indirectly inherited by neurons from germ cells. Nevertheless, it is intriguing to speculate on this possibility based on the paired evidence provided by our study, given specific neuronal genes and their predicted upstream regulatory miRNAs are significantly altered. A more comprehensive characterization of gene expression differences in F1 offspring brains, extending to the F2 generation, will be required in future. *Bdnf* and *Igf2* are well known to regulate anxiety behavior and fear extinction^29, 30, 78, 79, 80^ and are separately susceptible to glucocorticoid treatment.^11, 81^ Furthermore, on the basis of the strong male germline effect on behavior, *Igf2* was a prime gene target as it is a well-established paternally imprinted gene, and its expression was differentially modulated in male and female F1 offspring in this study. Previous human studies associated adverse maternal environmental conditions during early prenatal development with decreased levels of *Igf2* methylation,^29, 82^ implying a possible increase in *Igf2* gene expression.

The collective evidence presented here is an important step toward ultimately understanding the mechanisms involved in transmitting information about the paternal environment (specifically stress) across multiple generations, and has major evolutionary and public health implications. In summary, the emerging significance of the paternal environment in influencing the developmental trajectory of offspring behaviors and stress-related physiology has overarching implications for how we currently think about prenatal care. At present, periconceptual health advice is disproportionately focused on healthy lifestyle and diet in women. This now appears insufficient, as there is equivocal potential for the male to directly influence the health outcomes of the child. Future research to understand the consequences of paternal stress on both the male partner and his children will facilitate improved management of pre-conceptual stress in males and ultimately public health. As the burden of disease associated with mental health problems continues to escalate,^83^ there could be further major socioeconomic implications originating from our scope of research, which seeks to minimize the risk of future generations developing anxiety, depression and other stress-related mental health disorders.
