# Supplementary material for: Elevated paternal glucocorticoid exposure alters the small noncoding RNA profile in sperm and modifies anxiety and depressive phenotypes in the offspring
Source: Transl Psychiatry. 2016 Jun 14;6(6):e837–. doi: 10.1038/tp.2016.109 (PMC4931607; doi:10.1038/tp.2016.109)
Supplement: Supplementary Information [file tp2016109x1.docx]

**Supplementary methods**

Maternal behaviour

Three separate observations were taken each day: in the morning (10:00h), the early afternoon (14:00h) and before lights out (17:00h) for one hour each. Dams were observed for the following behaviours: licking/grooming, active nursing, passive nursing and nest building as nurturing behaviour; self-grooming and eating/drinking as self-maintenance behaviour; climbing/digging as neglecting behaviour. Individual behaviours were recorded and then the frequency of each of the types of behaviour was analysed. No maternal behaviour data was recorded on PND3 when ultrasonic vocalisation testing was conducted.

*Fear conditioning*

Separate groups of behaviourally naïve mice were subject to fear conditioning. 14-week old control and CORT-treated males, F1 offspring at PND15 +/- 1 day and 8-week old F1 offspring were tested as previously described ^28^. Chambers were rectangular (31.8 × 25.4 × 26.7 cm) (Med Associates, USA), with stainless steel grid floors with 36 rods (3.2 x 7.9 mm), equipped with a Med Associates VideoFreeze system (Med Associates, USA). Two different contexts were created as described previously ^28^. A constant-current shock generator was used to deliver electric shock (0.7mA, 1s) (unconditioned stimulus, US) to the floor of the chambers as required. A programmable tone generator, speaker and sound calibration package was used to deliver auditory tone (volume: 80 dB; frequency: 5000 Hz) (conditioned stimulus, CS).

Mice were placed in an experimental chamber for 790 s in total. During the first 2 min period baseline freezing measurement was collected. Following this, all animals received 6 tone–foot-shock (CS-US) pairings. Each pairing consisted of a 10s tone that co-terminated with a 1s shock. Inter-trial intervals (ITI) ranged from 85 to 135s, with an average of 110s. Freezing was calculated from the first 9s of each CS presentation to avoid confounding effects of the shock presentation on movement (as described in Handford et al. 2014). 2 minutes following the last presentation, the mouse was removed from the experimental chamber and placed back into the home cage.

The day following conditioning, mice were tested for their CS memory by being placed in a different context to that in which they were conditioned. Mice were allowed a 2 min period during which baseline freezing was measured. They then received 45 presentations of a 10s tone in the absence of the shock with ITI of 10s (i.e. extinction). Percentage freezing reported is based on 10s of tone blocked into average freezing of 15 tones to represent early, middle and late extinction. Mice received 2 days of extinction.

Primer Sequences

| Gene | Forward Primer | Reverse Primer |
| --- | --- | --- |
| Cycophillin | 5’ CCCACCGTGRRCTTCGACA 3’ | 5’ CCAGTGCTCAGAGCTCGAAA 3’ |
| GR | 5’ AGGCCGCTCAGTGTTTTTCTA 3’ | 5’ TACAGCTTCCACACGTCAGC 3’ |
| MR | 5’ GGCTTCTGGGTGTCACTATGG 3’ | 5’CACAGATAGTTGTGTTGTCCTTCCA 3’ |
| BDNF ex I | 5' CCTGCATCTGTTGGGGAGAC3' | 5' GCCTTGTCCGTGGACGTTTA 3' |
| BDNF ex IV | 5' CAGAGCAGCTGCCTTGATGTT 3' | 5' GCCTTGTCCGTGGACGTTTA 3' |
| BDNF total | 5' GCGCCCATGAAAGAAGTAAA 3' | 5' TCGTCAGACCTCTCGAACCT 3' |
| IGF2 | 5’ TCTACTTCAGCAGGCCTTCA 3’ | 5’ GAACTCGTCCGGAAGTACG 3’ |

Cyclophilin was used as the endogenous control. PCR conditions as follows: 50°C for 2 minutes, 95°C for 10 minutes, followed by 40x cycles of 95°C for 15 seconds and 60°C for 1 minute. Validation of miR targets was performed with Qiagen miRNA assays and U6, SNORD61, SNORD95, SNORD96A as control genes. Melt curve analysis was performed for all runs. The relative expression levels of target genes were determined using comparative Ct (ΔΔCt) method and normalised to the mean expression of the relevant control group.

*Physiological and molecular studies*

Mice were killed by cervical dislocation for tissue collection between 09:00 – 12:00H. Blood was collected through cardiac puncture and left to clot at room temperature for 30 minutes. Following centrifugation at 1100 x g for 15 minutes serum was collected and stored at -20°C. Serum corticosterone levels were determined with an EIA Kit (Cayman Chemical, MI, USA) in triplicate according to the manufacturer’s instructions. Post-stress samples were collected from mice immediately after forced-swim testing. Adrenal glands, testes, caudal epididymis and hippocampus were dissected, snap frozen in liquid nitrogen and stored at -80°C. For small RNA sequencing, mature spermatozoa were collected using the swim up method ^14^. In order to harvest sperm for small RNA sequencing, the caudal epididymis was dissected, then a single lateral cut was made with a surgical blade. This was immersed into 1%BSA/PBS and incubated at 37°C for at least 30 minutes. Epididymal tissue was removed before centrifugation for 10 minutes at 400 g. The supernatant was removed immediately and the pellets were homogenized by pipetting vigorously in QIAzol lysis reagent (QIAGEN, VIC, Australia) prior to storage at -80°C.

*Sequencing analysis normalisation*

Normalisation and statistical analysis on the count data was executed using EdgeR ^36^, keeping only those genes with > 10 counts per million (CPM) in *all* samples of at least one of the sample groups. Read depths were between 2.1M and 7.6M reads and all samples were included in the analysis with the exception of one sample, which had to be dropped due to a low sequence yield of only 0.2M reads. EdgeR adjusts the differential expression analysis for varying sequencing depths, such that all normalisation factors were between 0.59 and 1.38. The data was scaled using trimmed mean of M-values (TMM) ^37^ and differentially expressed genes between all treatment group (Benjamini–Hochberg false discovery rate (FDR) < 0.05). Annotation was added using the ensemble human gene annotation package bioMart ^38^.

*Sequencing analysis miRNA binding prediction*

The gene region was restricted to 2000bp upstream flanking region and the 3’UTR. The minimum seed length was set at 7 nucleotides, which is the minimum number of nucleotides of miRNA sequence for a target interaction. Predicted binding analysis was limited to the miRanda, miRWalk and TargetScan prediction algorithms and a target must be positively matched by at least two of these to be included.
