# Supplementary material for: Elevated paternal glucocorticoid exposure alters the small noncoding RNA profile in sperm and modifies anxiety and depressive phenotypes in the offspring
Source: Transl Psychiatry. 2016 Jun 14;6(6):e837–. doi: 10.1038/tp.2016.109 (PMC4931607; doi:10.1038/tp.2016.109)
Supplement: Supplementary Figure 1 [file tp2016109x2.docx]

**A
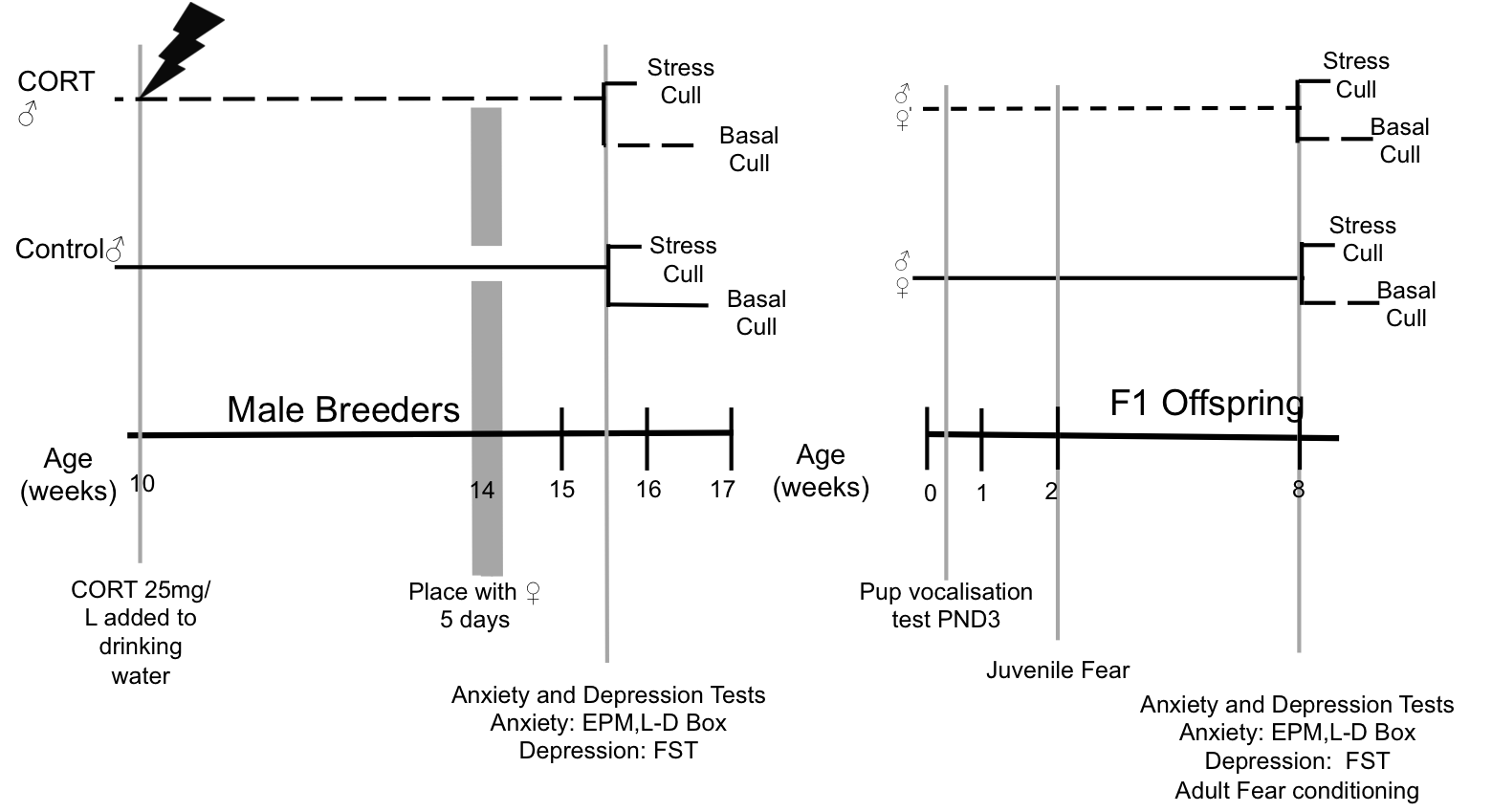
**

**B**

**
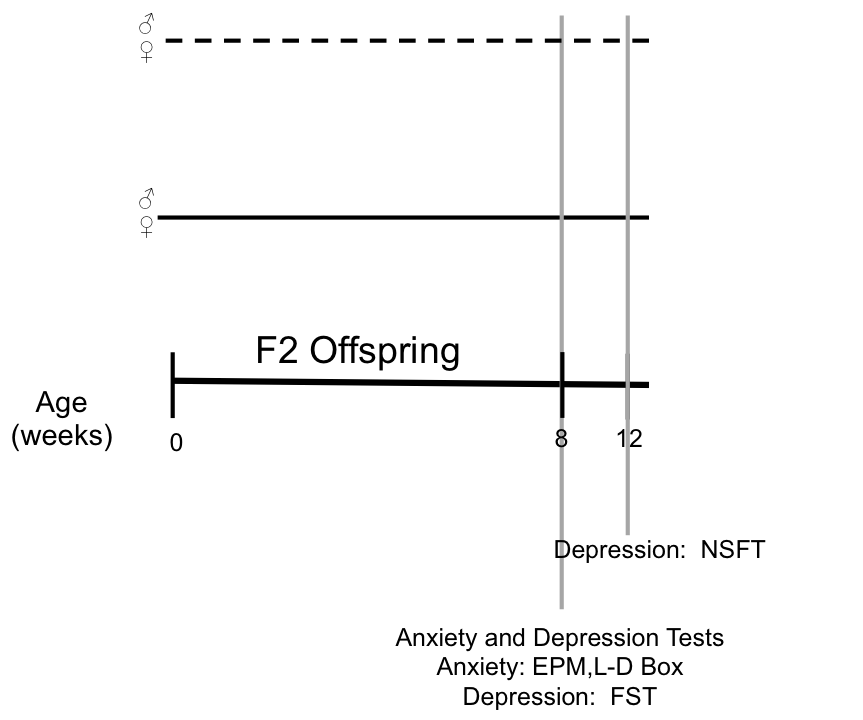
**

**Supplementary Figure S1. Study schematic across three generations.** A) Diagrammatic summary of the experimental procedures on F0 male breeders and the F1 offspring. B) Behavioural testing of the adult F2 offspring.
