# Supplementary material for: Elevated paternal glucocorticoid exposure alters the small noncoding RNA profile in sperm and modifies anxiety and depressive phenotypes in the offspring
Source: Transl Psychiatry. 2016 Jun 14;6(6):e837–. doi: 10.1038/tp.2016.109 (PMC4931607; doi:10.1038/tp.2016.109)
Supplement: Supplementary Figure 2 [file tp2016109x3.docx]

** Supplementary Figure S2. Effect of paternal CORT on female behaviour and litter size.**

There were no effects of exposure to CORT males on nurturing, self-maintenance or neglecting behaviours (A). Day 0 represents the morning the pups were born; no recordings were taken on Day 3 due to measuring vocalisations, n=12-18 litters per group, values represent means ± SEM. Throughout the entire study we observed no effect of paternal CORT on F1 litter size or sex ratio, number of pups from 26 control and 24 CORT litters (B). There were also no significant differences for F2 litters from 6 control and 7 CORT litters (C). Box plot whiskers represent minimum and maximum.
