# Supplementary material for: Elevated paternal glucocorticoid exposure alters the small noncoding RNA profile in sperm and modifies anxiety and depressive phenotypes in the offspring
Source: Transl Psychiatry. 2016 Jun 14;6(6):e837–. doi: 10.1038/tp.2016.109 (PMC4931607; doi:10.1038/tp.2016.109)
Supplement: Supplementary Figure 3 [file tp2016109x4.docx]

**Supplementary Figure S3. CORT-treatment did not affect male fertility.**

There were no effects of CORT on testes weights (A) and a representative histological section of testes showing seminiferous tubules of control (left) and CORT (right) mice (B). There were no effects of CORT on the daily sperm production count (C) and a representative histological sample of control (left) and CORT (right) sperm (D). Data represents means ± SEM.
