# Supplementary material for: Elevated paternal glucocorticoid exposure alters the small noncoding RNA profile in sperm and modifies anxiety and depressive phenotypes in the offspring
Source: Transl Psychiatry. 2016 Jun 14;6(6):e837–. doi: 10.1038/tp.2016.109 (PMC4931607; doi:10.1038/tp.2016.109)
Supplement: Supplementary Figure 4 [file tp2016109x5.docx]

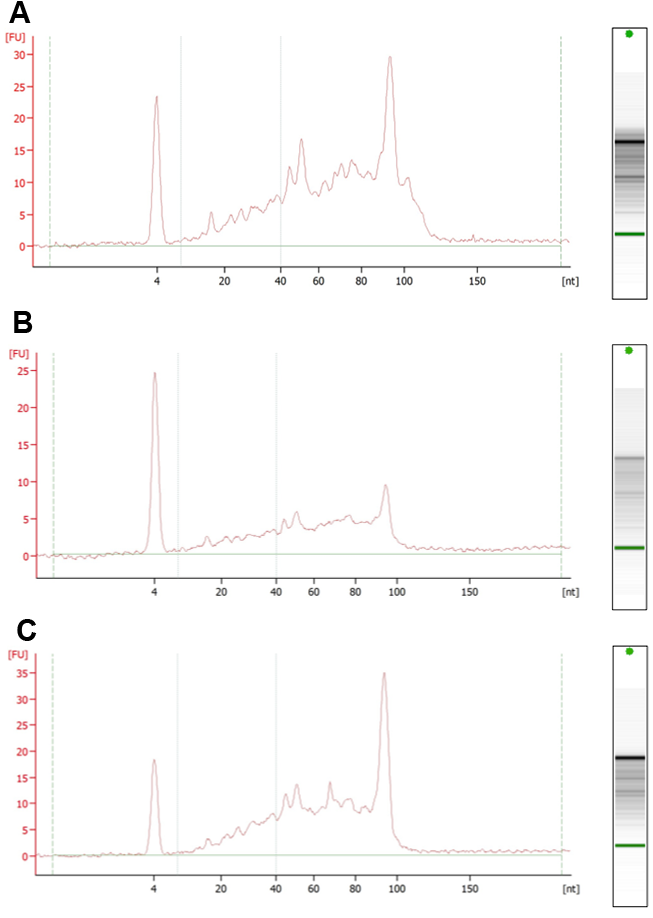
**Supplementary Figure S4. Representative bioanalyser result from sperm small RNA.**

Electropherograms obtained from the Agilent Bioanalyser 2100 using the small RNA kit showing Fluorescent Units (FU) per nucleotide (nt). Representative sperm samples from control animals (**A**) and CORT animals (**B**). Samples are pools of 4 animals per group.
