# Supplementary material for: Elevated paternal glucocorticoid exposure alters the small noncoding RNA profile in sperm and modifies anxiety and depressive phenotypes in the offspring
Source: Transl Psychiatry. 2016 Jun 14;6(6):e837–. doi: 10.1038/tp.2016.109 (PMC4931607; doi:10.1038/tp.2016.109)
Supplement: Supplementary Table 1 [file tp2016109x6.docx]

| **Gene name** | **CHR** | **Size** | **Fold change (log2)** | **Counts per million** | **p** | **FDR** |
| --- | --- | --- | --- | --- | --- | --- |
| Gm24148 | 1 | 80 | -2.042 | 8.923 | 0.001 | 0.011 |
| chr6.tRNA1016-CysGCA | 6 | 72 | -2.128 | 3.876 | 0.000 | 0.007 |
| Gm4419 | 12 | 1729 | -2.296 | 3.587 | 0.000 | 0.002 |
| chr3.tRNA303-GluCTC | 3 | 72 | -2.642 | 12.323 | 0.000 | 0.002 |
| chr1.tRNA709-GluCTC | 1 | 72 | -2.659 | 12.331 | 0.000 | 0.002 |
| chr10.tRNA90-GluCTC | 10 | 72 | -2.669 | 12.323 | 0.000 | 0.002 |
| Mir144 | 11 | 66 | 4.057 | 8.819 | 0.000 | 0.002 |
| Mir451a | 11 | 72 | 3.824 | 11.728 | 0.000 | 0.002 |
| Mir190b | 3 | 80 | 2.972 | 5.026 | 0.000 | 0.006 |
| Mir98 | X | 108 | 2.735 | 5.948 | 0.000 | 0.006 |
| Mir194-1 | 1 | 67 | 2.584 | 6.350 | 0.000 | 0.004 |
| Mir669a-1 | 2 | 97 | 2.502 | 3.206 | 0.000 | 0.002 |
| Mir192 | 19 | 89 | 2.482 | 11.343 | 0.000 | 0.009 |
| Mir301 | 11 | 86 | 2.417 | 7.565 | 0.002 | 0.022 |
| Mir742 | X | 65 | 2.363 | 5.514 | 0.000 | 0.011 |
| Mir881 | X | 78 | 2.354 | 11.698 | 0.001 | 0.011 |
| Mirlet7i | 10 | 90 | 2.353 | 9.492 | 0.001 | 0.011 |
| Mir21a | 11 | 92 | 2.326 | 11.925 | 0.001 | 0.018 |
| Mir362 | X | 65 | 2.320 | 4.482 | 0.000 | 0.011 |
| Mir350 | 1 | 99 | 2.314 | 6.917 | 0.000 | 0.010 |
| Mir194-2 | 19 | 86 | 2.309 | 6.673 | 0.000 | 0.010 |
| Mir30a | 1 | 82 | 2.304 | 13.361 | 0.001 | 0.017 |
| Mir26b | 1 | 85 | 2.302 | 9.856 | 0.000 | 0.011 |
| Mir669a-6 | 2 | 97 | 2.289 | 3.288 | 0.000 | 0.006 |
| Mirlet7g | 9 | 88 | 2.286 | 10.420 | 0.001 | 0.011 |
| Mir449a | 13 | 91 | 2.252 | 9.368 | 0.000 | 0.011 |
| Mir467e | 2 | 87 | 2.238 | 5.133 | 0.000 | 0.011 |
| Mir883b | X | 78 | 2.208 | 7.391 | 0.000 | 0.011 |
| Mir471 | X | 67 | 2.202 | 7.770 | 0.001 | 0.019 |
| Mir141 | 6 | 72 | 2.196 | 12.929 | 0.002 | 0.022 |
| Mir93 | 5 | 88 | 2.191 | 11.089 | 0.001 | 0.014 |
| Mir340 | 11 | 98 | 2.187 | 9.081 | 0.000 | 0.011 |
| Mir32 | 4 | 70 | 2.130 | 5.487 | 0.001 | 0.019 |
| Gm24111 | 13 | 97 | 2.108 | 10.443 | 0.002 | 0.022 |
| Mir743b | X | 77 | 2.105 | 11.875 | 0.001 | 0.017 |
| Mir669a-11 | 2 | 97 | 2.099 | 3.393 | 0.000 | 0.010 |
| Mir101b | 19 | 97 | 2.087 | 7.298 | 0.001 | 0.019 |
| Mir374b | X | 95 | 2.082 | 5.901 | 0.001 | 0.018 |
| Mirlet7f-2 | X | 83 | 2.077 | 10.578 | 0.002 | 0.022 |
| Mir146 | 11 | 65 | 2.065 | 10.682 | 0.004 | 0.034 |
| Mir872 | 4 | 81 | 2.061 | 8.765 | 0.001 | 0.011 |
| Mir9-3 | 7 | 90 | 2.055 | 4.474 | 0.002 | 0.022 |
| mt-Ts2 | MT | 59 | 2.045 | 7.242 | 0.005 | 0.034 |
| Mir195a | 11 | 94 | 2.043 | 7.856 | 0.001 | 0.021 |
| Mir146b | 19 | 109 | 2.038 | 7.891 | 0.005 | 0.036 |
| Mir34c | 9 | 77 | 2.031 | 13.672 | 0.003 | 0.025 |

Supplementary Table 1. Genes with 2-fold or greater change in expression in CORT sperm.
